# Assessment of the interaction of Portland cement-based materials with blood and tissue fluids using an animal model

**DOI:** 10.1038/srep34547

**Published:** 2016-09-29

**Authors:** P. Schembri Wismayer, C. Y. K. Lung, F. Rappa, F. Cappello, J. Camilleri

**Affiliations:** 1 Department of Anatomy, Faculty of Medicine and Surgery, University of Malta, Malta; 2 Dental Materials Science, Faculty of Dentistry, The University of Hong Kong, Hong Kong; 3 Department of Experimental Biomedicine and Clinical Neurosciences, University of Palermo, Palermo, Italy; 4 Department of Restorative Dentistry, Faculty of Dental Surgery, University of Malta, Malta.

## Abstract

Portland cement used in the construction industry improves its properties when wet. Since most dental materials are used in a moist environment, Portland cement has been developed for use in dentistry. The first generation material is mineral trioxide aggregate (MTA), used in surgical procedures, thus in contact with blood. The aim of this study was to compare the setting of MTA *in vitro* and *in vivo* in contact with blood by subcutaneous implantation in rats. The tissue reaction to the material was also investigated. ProRoot MTA (Dentsply) was implanted in the subcutaneous tissues of Sprague-Dawley rats in opposite flanks and left *in situ* for 3 months. Furthermore the material was also stored in physiological solution *in vitro*. At the end of the incubation time, tissue histology and material characterization were performed. Surface assessment showed the formation of calcium carbonate for both environments. The bismuth was evident in the tissues thus showing heavy element contamination of the animal specimen. The tissue histology showed a chronic inflammatory cell infiltrate associated with the MTA. MTA interacts with the host tissues and causes a chronic inflammatory reaction when implanted subcutaneously. Hydration *in vivo* proceeds similarly to the *in vitro* model with some differences particularly in the bismuth oxide leaching patterns.

Portland cement is used in the construction industry as a binder in concrete. Its main feature is its hydraulic nature as the material sets and develops its properties in the presence of moisture. Materials based on industrial Portland cement have been introduced in dentistry. The first generation material was mineral trioxide aggregate (MTA) which is composed of Portland cement and bismuth oxide^1^. The Portland cement component when mixed with water results in the formation of calcium silicate hydrate, calcium hydroxide, and ettringite^2^. The bismuth oxide is added to enhance the material radiopacity. All materials implanted in the human body need to be visible on a radiograph. MTA has several uses in dentistry with its main application being used as a root-end filler or perforation repair material in surgical procedures where it is immediately contaminated with blood. When in clinical use the calcium hydroxide, which is a by-product of material hydration reacts with phosphates present in physiological fluids forming hydroxyapatite^3^,^4^. This is postulated to be the reason for the material success.

MTA is immediately contaminated with blood as soon as placed in surgical sites. The contamination of MTA by blood has been investigated in a number of laboratory studies in terms of the effect on its physical properties and material microstructure. There is little doubt that blood contamination on the surface of MTA and in particular, when incorporated into the material is detrimental to material hydration and thus ultimate physical properties and performance. Unfortunately, MTA is often placed in contact with vital tissues that ooze blood/serum (pulp capping) or in situations where blood pools on its surface (root-end filling) and the impact of blood contamination on the material properties is important.

MTA exhibited a retarded setting time in the presence of blood^5^. Mixing MTA with blood has a negative effect on its surface hardness, microstructure^6^ and compressive strength^7^. Essentially, it has been reported that when blood becomes incorporated into MTA, its compressive strength is reduced with the result that in clinical situations in which blood becomes mixed with MTA, its physical properties are likely to be compromised^7^. Even addition of accelerators which should enhance the material properties do not improve the material characteristics in contact with blood^8^. Similar effects have been reported when foetal bovine serum was used to contaminate the surface of MTA^9^.

The hydration state of MTA mixed with blood has also been reported^10^. Specimens partially mixed with blood were more completely hydrated than those mixed entirely with blood. All specimens hydrated in the presence of blood were less hydrated at the same time period than specimens hydrated completely with water. Lack of formation of the crystalline calcium hydroxide in the early stage of the hydration process was noted together with the absence of acicular crystals, characteristic of ettringite crystals. Rounded crystals present on the MTA surface and lack of needle-like crystals were observed when MTA was assessed after contact with blood or synthetic tissue-fluid. Maximum gap width and gap perimeter in the blood-exposed group were significantly larger than those in the synthetic tissue fluid-exposed group^11^.

All the investigations have been performed *in vitro*. Blood and blood products cannot be maintained fresh throughout the period of experimentation. Thus the *in vivo* implications are questionable. The clinical performance of MTA is based on the assumption that the material hydrates and calcium hydroxide is produced after the material has set. The interaction of calcium hydroxide with physiological fluids has only been investigated *in vitro*. The deposition of apatite on the material surfaces is hypothetical. The *in vitro* studies all indicate that contact with blood modifies the material microstructure and interferes with setting. The aim of this study is to investigate the MTA hydration in an animal model and to correlate the hydration status with histological findings.

## Results

All the animals tested were in good health and the areas of subcutaneous implantation had healed well. Less MTA than that implanted in the subcutaneous tissues was retrieved indicating some reabsorption. Furthermore in some of the animals no material was visible in the subcutaneous tissue. In fact histology was performed in 4 animals and material characterization in three.

### Tissue histology and elemental mapping

**Figure 1:**
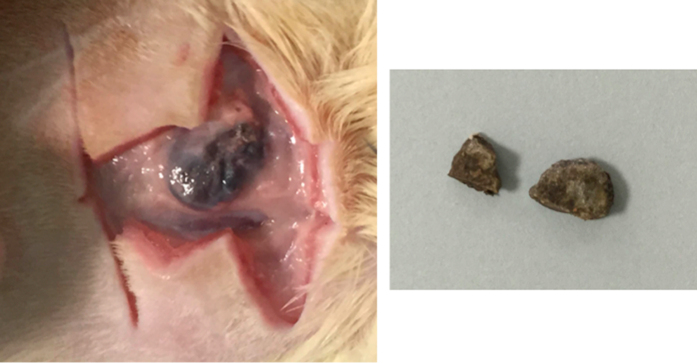
ProRoot MTA implanted subcutaneously in rats showing dark brown discoloration, which was more evident after retrieval.

Retrieval of material from subcutaneous implantation sites is shown in Fig. 1. The ProRoot MTA caused a black hue, which was observed in all the samples retrieved from the subcutaneous tissues (Fig. 1). In a few cases the material was lying deeper so the colour change could only be observed after the material was dissected out.

**Figure 2:**
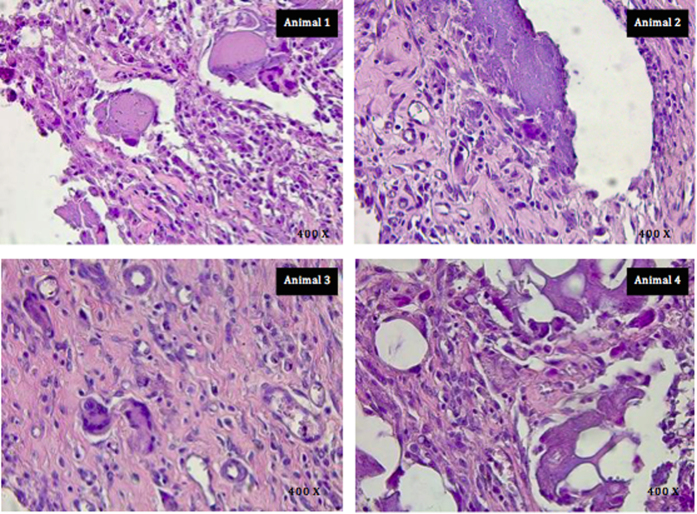
Light micrographs at 400X magnification representative of the findings in each animal tested showing the chronic inflammatory cell infiltrate associated with ProRoot MTA in subcutaneous implantation in the rat model. (Staining Hematoyxlin and Eosin).

The tissue histology is shown in Fig. 2. The histology was performed on 4 specimens. In one case the MTA could not be retrieved. Some reabsorption was observed with all the specimens but in one case no material was found in the subcutaneous tissues. In all animals, in the subcutaneous tissue, there was necrotizing granulomatous reaction (granulomatous foreign body reaction). As in a typical foreign-body reaction, there is a chronic cell infiltrate with histiocytes and multinucleated giant cells around the material implanted that appeared eosinophilic. In addition lymphocytes were present as well as plasma cells and granulocytes. There were other features, which included cystic areas containing necrotic material surrounded by histiocytes, giant cells and leucocytes (Animal 2 in Fig. 2). At the bottom of the sample (in proximity of the deep margin) there were numerous ovoid cavities varying in size within the implanted eosinophilic material. These cavities are remnants of processing and were surrounded by an inflammatory infiltrate of macrophages and lymphocytes resulting in the typical “Swiss cheese” appearance (Animal 4 in Fig. 2). Finally there was a moderate angiogenesis and mild fibrosis (Animal 2 and 3 in Fig. 2).

**Figure 3:**
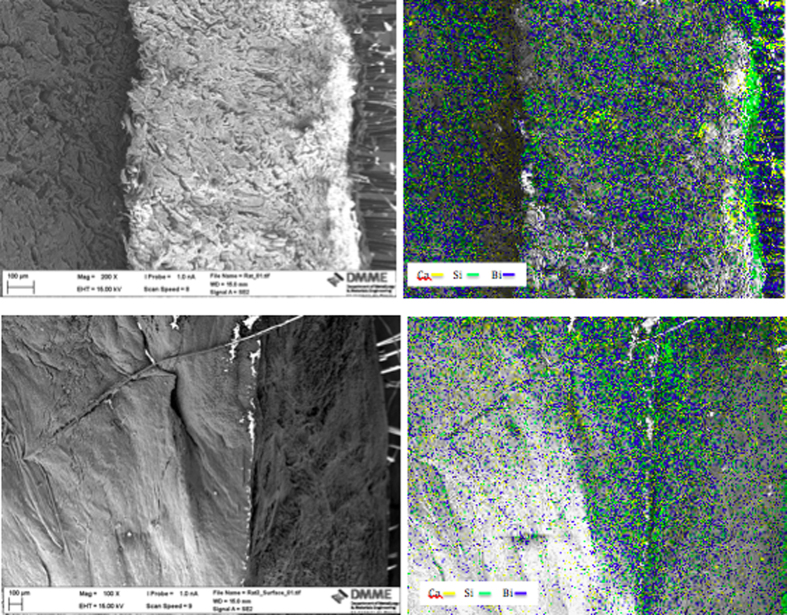
Scanning electron micrograph of transverse sections through the rat skin and subcutaneous tissues in contact with the ProRoot MTA showing elemental maps for calcium, silicon and bismuth overlapped on the micrograph.

The scanning electron micrograph and elemental maps for calcium, silicon and bismuth are shown in Fig. 3. For all samples prepared for electron microscopy and mapping, traces of both calcium and silicon were visible just underneath the skin where the material was in contact with the subcutaneous tissues. The bismuth map showed extensive contamination of all the tissues.

### Material characterization

**Figure 4:**
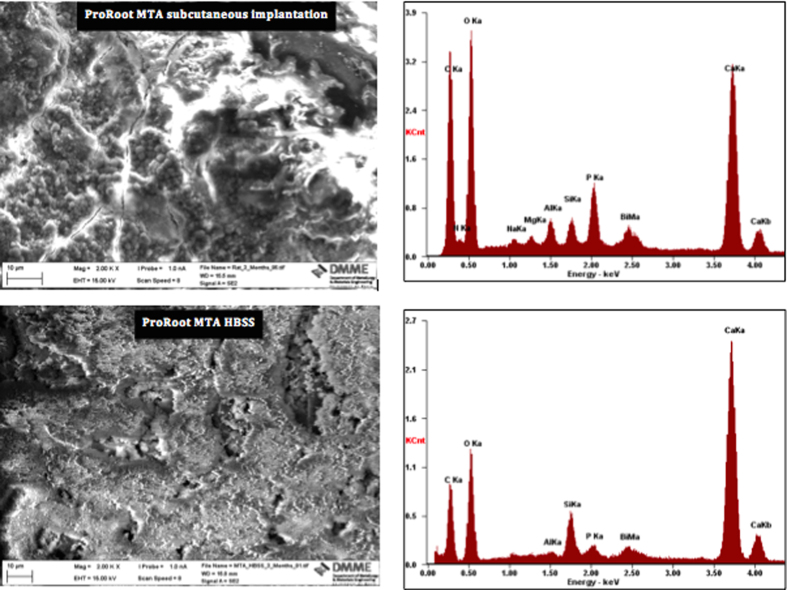
Surface scanning electron micrographs and energy dispersive spectroscopy plots of ProRoot MTA in contact with blood retrieved from subcutaneous implantation sites, and also in contact with Hank’s balanced salt solution for 3 months.

**Figure 5:**
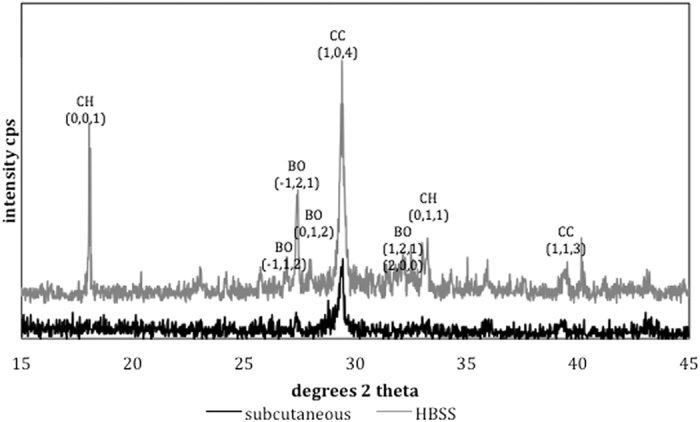
Surface X-ray diffraction plots of ProRoot MTA retrieved from subcutaneous implantation site and in contact with Hank’s balanced salt solution for 3 months (BO: bismuth oxide, CH: calcium hydroxide, CC: calcium carbonate).

The surface SEM/EDS analysis and XRD scans are shown in Figs 4 and 5 respectively. The surface of the material retrieved from subcutaneous implantation sites exhibited a dense coating, which was rich in calcium and phosphorus. Globular crystal deposits were also evident. These globular deposits were composed of calcium and phosphorus. The MTA in contact with HBSS exhibited hydrating cement particles. Deposition of globular crystals rich in calcium and phosphorus were observed over the cement particles. The indexed XRD surface plots are shown in Fig. 5. The ProRoot MTA from subcutaneous implant and that in contact with HBSS exhibited a peak at 29.46°2θ, which was of lower intensity for the implanted MTA. This peak matched calcium carbonate (ICDD: 01-080-2794). In addition the MTA soaked in HBSS exhibited addition peaks for calcium hydroxide at 18.07 and 34.10°2θ (ICDD: 01-076-0571) and bismuth oxide with the main peak at 27.48°2θ (ICDD: 04-008-7767).

The bulk material characteristics were observed on SEM/EDS of polished sections, powder diffractometry and FT-IR spectroscopy. The back-scatter scanning electron micrographs of the polished ProRoot MTA in contact with different environments for 3 months are shown in Fig. 4. The ProRoot MTA in contact with blood in subcutaneous tissues exhibited some unhydrated cement particles but was mostly composed of reaction by-product. The MTA stored in HBSS was more completely hydrated with less unreacted cement particles present. For both environments a small phosphorus peak was present in the material matrix together with particles rich in bismuth.

**Figure 6:**
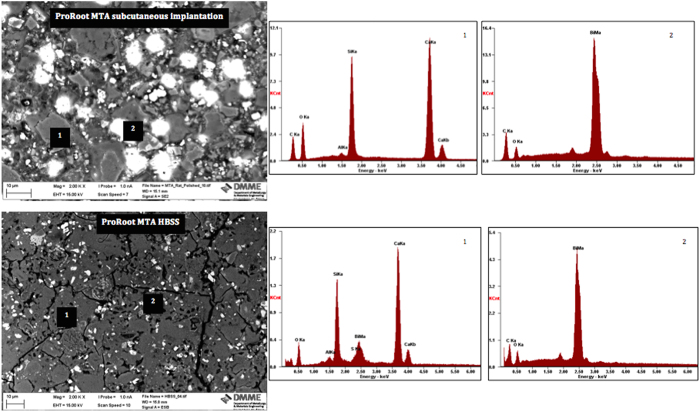
Back scatter electron scanning electron micrographs of polished sections of ProRoot MTA retrieved from subcutaneous implantation site and in contact with Hank’s balanced salt solution for 3 months.

**Figure 7:**
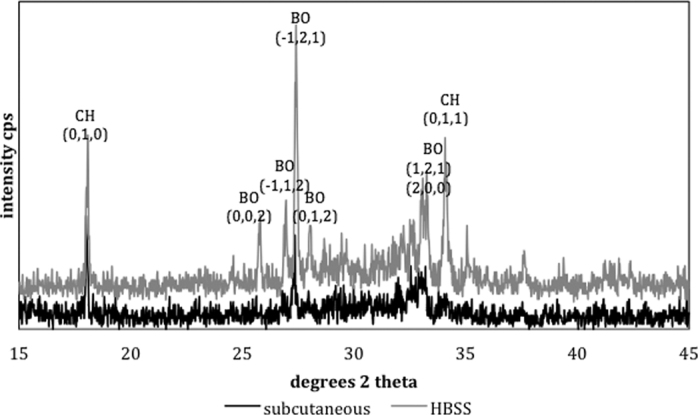
Powder X-ray diffraction plots of ProRoot MTA in contact with blood from specimens retrieved from subcutaneous implantation sites and Hank’s balanced salt solution for a period of 3 months. (BO: bismuth oxide, CH: calcium hydroxide).

The indexed powder X-ray diffraction plots and FT-IR spectra of ProRoot MTA powder and in different environments are shown in Figs 6 and 7. The set MTA exhibited peaks for bismuth oxide and calcium hydroxide with the tricalcium silicate peaks (32 and 34°2θ) flattening due to material reaction. The MTA retrieved from subcutaneous tissue exhibited less bismuth oxide peaks with only the main peak at 27.48°2θ being visible. In both environments calcium hydroxide peak at 18.07°2θ was observed.

**Figure 8:**
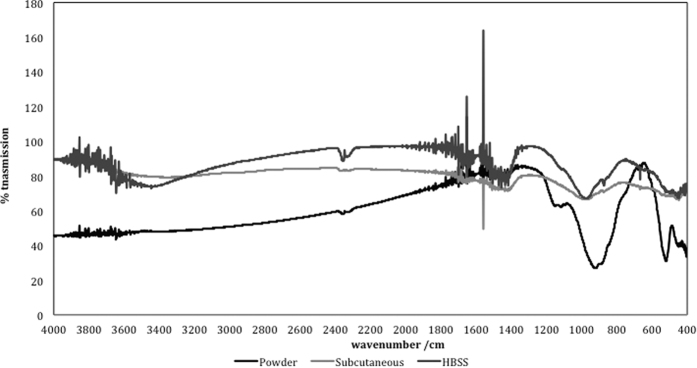
FT-IR plots of ProRoot MTA in different environmental conditions for a period of 3 months.

The FT-IR plots are shown in Fig. 8. The un-hydrated MTA displayed a tricalcium silicate peak in the low frequency region at ~500–550, ~800–830, ~850–870, ~920–950 cm^−1^ which is caused by the presence of silicate functional groups. This was also identified in the set materials for both environmental conditions but the peaks were less intense indicating the reaction of the tricalcium silicate. The peaks at ~1100–1150 cm^−1^ are caused by the presence of sulphate ions in calcium sulphate as ProRoot MTA is a Portland type cement. The small peak at ~1400–1450 cm^−1^ was due to carbonate which indicated some carbonation of the powder in atmospheric conditions. Both the material retrieved from subcutaneous implantation and that in contact with HBSS showed a lot of noise at ~1400–1450 cm^−1^ followed by a sharp peak. This shows the presence of the carbonate group. The MTA in HBSS only showed the presence of phosphate group ~600 cm^−1^ and ~950–1000 cm^−1^, hydroxyl ions at ~650 cm^−1^ and carbonate from carbonated apatite at ~850–870 cm^−1^. Thus while the material from the subcutaneous implantation showed the presence of carbonation, the MTA in contact with HBSS showed presence of carbonated apatite. The formation of specific phases is dependent on the availability of specific ions in solution.

**Figure 9:**
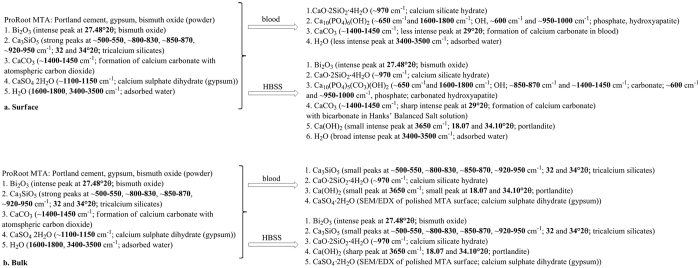
Reaction scheme of setting of ProRoot MTA under different environments: (**a)** surface and (**b)** bulk.

All the set materials exhibited a peak in the region of ~970 cm^−1^ which is assigned to calcium silicate hydrate. Also a lot of noise in the high frequency region at 3600–3800 cm^−1^ and between 1600 and 1800 cm^−1^, which indicates the presence of free OH^−^ ions was observed. A small sharp peak at 3660 cm^−1^ detected in the MTA powder and MTA in HBSS is assigned to OH stretching in Ca(OH)_2_. In the unhydrated material this is due to material pre-hydration in air. The peaks intensified in the hydrated material in all environments due to both physically adsorbed water and also OH^−^ ion formation during cement hydration. The setting of surface and bulk MTA under different environments is summarized by a reaction scheme based on the characterization results (Fig. 9).

### Ion leaching analysis

The results for the leaching in both water and HBSS are shown in Table 1. Leaching of calcium, silicon and bismuth was higher in HBSS. The phosphorus concentration was depleted indicting uptake of phosphorus from the solution. The MTA was thus reacting with the phosphorus present in the HBSS.

## Discussion

This study investigated the hydration of ProRoot MTA *in vivo* using an animal model, which was compared to material hydration *in vitro*. Tissue histology was also performed from subcutaneous implantation. Comparison of the present findings with previous research on MTA was thus possible and identification of the chemistry of MTA that was causing the specific changes was performed. ProRoot MTA was chosen for this study as it is the first MTA, which has been available for clinical use and a number of studies have been performed on this material. Both the surface and the bulk material characteristics were investigated since crystal deposition on the material surfaces may not necessarily imply changes in the bulk material. Furthermore various methods of characterization were employed in order to enable a broader picture of the material chemistry. The scanning electron microscopy and EDS analyses show material microstructure and elemental analyses. The specific elemental analyses enabled phase identification by X-ray diffraction analyses. The FT-IR spectroscopy identified the chemical groups present in the materials thus completing the chemical analyses.

The surface characteristics were different to the hydration of the bulk material. Bulk testing showed that for both the subcutaneous implanted material and material stored in HBSS there was the formation of calcium hydroxide. This indicated that material hydration occurred even when MTA was in contact with blood. This is in contrast to previous research showing the lack of hydration of MTA in contact with blood^6^,^10^. The reaction proceeded well as the unhydrated cement particles were coated by hydration product, which was also present in the material matrix as shown by the scanning electron micrographs of the polished samples. The difference between the *in vivo* and *in vitro* immersion in HBSS was the bismuth oxide. In the animal model the crystalline phase of bismuth was weak indicating that there was less bismuth oxide present in the material when compared to HBSS exposure. It is postulated that in the MTA in contact with blood, there is higher leaching of bismuth ions, which are reabsorbed by the tissues in the living organism. The leaching pattern of MTA was investigated by ICP analyses. In HBSS there was significantly more leaching of both bismuth and calcium when compared to water. Testing the leaching in the animal model was not possible. However the tissues in contact with the MTA showed a high bismuth contamination as verified by the elemental mapping. The distribution of bismuth in the subcutaneous tissues was more extensive than calcium and silicon. This finding further corroborates the reduction in bismuth oxide content in set MTA after 28 days of hydration as shown by Reitveld X-ray diffraction analyses reported in a previous study^2^.

The surface characteristics indicated the formation of a carbonate rich phase. This phase was only visible on the material surface and not in the material bulk. The surface characteristics are important, since cells and tissues are in contact with the material surfaces, thus any changes in material chemistry affect the cellular and tissue reactions. In the animal model no calcium hydroxide formation was observed as opposed to the MTA in contact with HBSS. Furthermore the phases present in the animal model were poorly crystalline. These changes were also observed in a study on explanted material retrieved from a failed apicectomy^12^. Only a peak at 29°2θ was observed on the explanted material and this was indicative of the presence of calcium carbonate. In this study the changes observed in the material stored in HBSS were different with calcium hydroxide formation on the material surface^12^. It has been postulated that the formation of specific phases depends on the ion availability of the surrounding tissues. The poorly crystalline carbonate phase indicates material reaction with carbon dioxide present in the tissues. Some phosphates were observed in the FT-IR scans of the MTA in contact with HBSS. This may indicate the presence of non-crystalline phosphates that cannot be identified by XRD analyses.

This is the first study that characterized the material and reports the tissue histology associated with the characterized MTA. Although MTA is used in contact with tooth and bone, the first line method of testing is subcutaneous implantation in the rat. A study on MTA implanted in the alveolus compared to subcutaneous implantation showed no difference in the tissue reaction with similar histology and more mature healing noted in a 30 day period compared to 7 day tissue contact^13^. Another study, which used two implantation sites namely subcutaneous and intraosseous showed that reactions to intraosseous implants of MTA were less intense than with subcutaneous implantation. Osteogenesis occurred in association with intraosseous implants indicating osteoconductivity but osteoindictivity was not demonstrated^14^.

Furthermore, the set materials were placed in polyethylene tubes following ISO standards for testing (ISO 10993-6:2009)^15^. The ISO specifies the use of polyethylene tubes for powders not for set materials. However most researchers still implant the test materials in the tubes and assess the tissue histology on a limited area of contact. In the current study, the MTA was implanted directly in contact with the subcutaneous tissues in order to be able to study the effect of blood contamination and complete tissue contact. The material retrieved was discoloured (Fig. 1). The discoloration has never been noted in previous studies where tissue histology was performed, however discoloration induced by blood contact has been reported^16^.

The tissue histology indicated the presence of a chronic cell infiltrate at 3 months of tissue contact. This is in accordance to previous findings by other researchers^13^,^14^,^17^,^18^,^19^. The tissue reactions were studied at early ages (7 days) with the maximum of 60 days contact^14^,^17^,^18^,^19^,^20^,^21^. Only one study evaluated longer contact time with 90-day histological assessment carried out^22^. In the current study the tissue reactions were evaluated after a 90-day implantation period similar to previous research^22^. Improvement in the inflammatory response could not be evaluated since shorter time frames were not assessed; this was not the scope of the study. Although most studies reported an improvement in the inflammatory reaction a chronic cell infiltrate was always invariably present with or without the presence of a fibrous capsule. An improvement in tissue inflammatory reaction is beneficial but in all cases of MTA subcutaneous tissue implantation, a long-term chronic cell infiltrate and a chronic tissue reaction to a foreign body was always observed. This is not what should be desirable with respect to adequate material tissue reaction.

## Methods

ProRoot MTA (Dentsply Tulsa Dental Specialities, Tulsa, OK, USA) was mixed at water to powder ratio of 0.35. The material was placed in contact with different media and was investigated after 3-month incubation. The media included blood contamination by subcutaneous implantation in rats and also placement in Hank’s balanced salt solution (HBSS; H6648, Sigma Aldrich, St. Louis, MO, USA) for 3 months. Material characterization after contact with the media was performed together with leachate analysis and tissue histology.

### Animal study

Five Srague-Dawley female rats with an initial weight of ranging from 300–400 g were used. The necessary approval for animal use was obtained by the Research Ethics Committee, at the University of Malta. The animal testing conformed to the NIH Guide for Care and Use of Laboratory Animals guidelines. Each animal received two identical implants of the freshly mixed mineral trioxide aggregate (ProRoot MTA; Dentsply Dental Specialities, Tulsa, OK, USA) in subcutaneous pockets created in contact with the connective tissue in the flanks. The rats were anesthetized by an intraperitoneal injection of Ketamine (90 mg/kg) and Xylazine (10 mg/kg). After anesthesia, the back was shaved and the skin cleansed with alcohol. All surgical procedures were done under aseptic conditions.

For the subcutaneous tissue implantation two incisions on either side of the midline of the rat’s back were made. A pocket underneath the skin was created by blunt dissection and the freshly mixed MTA was placed in contact with the connective tissue. The skin was sutured with 3-0 silk sutures.

All animals received daily paracatamol (150 mg/kg) and Ibuprofen (30 mg/kg) orally ad libitum in drinking water for 4 days after surgery. The animals were euthanized after 3 months. After euthanasia, on one side the tissues complete with the materials were dissected out and fixed in buffered 10% formaldehyde solution followed by decalcification in ethylene diamine tetracetic acid (EDTA).

#### Histological analysis

After decalcification all the specimens were paraffin-embedded to prepare them for histological analysis. Sections of 5 to 6 μm thickness were cut from paraffin blocks, by a microtome. These sections were deparaffinised with xylene for 10 minutes at 60 °C and hydrated with a decreasing ethanol gradient and then stained with haematoxylin eosin for histological examination using an automated Leica DM 5000 B microscope (Leica, Milan Italy) connected to a Leica DC 300 F camera (Leica, Milan, Italy). The specimens were randomly coded and examined by the pathologist for material identification. The samples were viewed under the light microscope and images were captured at different magnifications. The material from the other flank was retrieved, dried and prepared for characterization.

#### Scanning electron microscopy and elemental mapping

Three other rats also received subcutaneous implantation of MTA. After the 3-month interval the tissues in contact with the material were fixed, dried with ascending grades of ethanol, critically point dried, sectioned transversely and were then mounted on an aluminium stub and coated with gold. The tissues were viewed under the scanning electron microscope (Zeiss MERLIN Field Emission SEM, Carl Zeiss NTS GmbH, Oberkochen, Germany) at low magnification in order to visualize the skin and subcutaneous layer. Elemental maps for calcium, silicon and bismuth were acquired.

### Material characterization

The MTA derived from the subcutaneous tissue in the rat and the MTA in contact with HBSS for 3 months (n = 3) were characterized by scanning electron microscopy (SEM), energy dispersive spectroscopy (EDS), X-ray diffraction analyses and Fourier transform infrared (FT-IR) spectroscopy. Both surface analyses and bulk assessment was performed. For surface analyses the materials once retrieved were dried in a vacuum desiccator, carbon coated and then assessed by secondary electron imaging and EDS in the SEM. Phase analysis was performed by X-ray diffraction. The diffractometer (Bruker D8 Advance, Bruker Corp., Billerica, MA, USA) used Cu Kα radiation at 40 mA and 45 kV and the detector was rotated between 15–45° with a step of 0.02°2θ and a step time of 0.6 degrees/min. The sample holder was spun at 15 rpm. Phase identification was accomplished using search-match software utilizing ICDD database (International Centre for Diffraction Data, Newtown Square, PA, USA) where the specific phases were indexed and identified.

Bulk material analysis was performed by SEM and EDS of polished specimens, powder X-ray diffractometry and FT-IR spectroscopy. For SEM the materials were dried and impregnated in resin (Epoxyfix, Struers GmbH, Ballerup, Denmark) and the resin blocks were then ground and polished using an automatic polisher (Tegramin 20, Struers GmbH, Ballerup, Denmark). The specimens were mounted on aluminum stubs, carbon coated and viewed under the SEM in back-scatter mode at 2K and 5K magnifications. EDS analysis was also performed. For X-ray diffraction analysis and FT-IR spectroscopy MTA specimens were dried and crushed into a very fine powder using an agate mortar and pestle. Phase analysis was performed using the same settings described previously. FT-IR was performed on the powdered specimens by preparing KBr discs made up of two to five milligrams of test material ground with 100 mg potassium bromide forming a pellet and tested by FT-IR spectroscopy (IRAffinity-1; Shimadzu, Kyoto, Japan) over the range 400–4000 cm^−1^.

### Ion leaching analysis

Cylindrical specimens 10 mm in diameter and 2 mm thick were prepared of ProRoot MTA mixed at a water to powder ratio of 0.35. The materials were allowed to set for 24 hours at 37 °C and 100% humidity. Specimens were removed from the moulds, weighed and immersed in either water or HBSS for 3 months. The calcium ion leaching was assessed using inductively coupled plasma (ICP) spectroscopy.

## Conclusions

The surface and bulk properties of ProRoot MTA were affected when setting in different environments, blood and HBSS. Hydration process is similar between *in vivo* and *in vitro* models but some hydration products are different. The interaction of MTA implanted subcutaneously caused a chronic inflammatory reaction. Bismuth was leached in solution and was also shown to be contaminating the tissues.

## Additional Information

**How to cite this article**: Schembri Wismayer, P. *et al.* Assessment of the interaction of Portland cement-based materials with blood and tissue fluids using an animal model. *Sci. Rep.* **6**, 34547; doi: 10.1038/srep34547 (2016).

## Figures and Tables

**Table 1:** Concentration of elements leached from ProRoot MTA in water and Hank’s balanced salt solution (HBSS).

Material	Soaking solution	Concentration of element mg/g			
		Calcium	Silicon	Bismuth	Phosphorus
ProRoot MTA	Water	13,049	4	34	0
	HBSS	20,173	41	354	−98

## References

[b1] Torabinejad M. & White D. J. Tooth filling material and method of use. US Patent 5,769,638 (1995).

[b2] Camilleri J. Characterization of hydration products of mineral trioxide aggregate. Int Endod J. 41, 408–417 (2008). 18298574 10.1111/j.1365-2591.2007.01370.x

[b3] Sarkar N. K., Caicedo R., Ritwik P., Moiseyeva R. & Kawashima I. Physicochemical basis of the biologic properties of mineral trioxide aggregate. J Endod. 31, 97–100 (2005). 15671817 10.1097/01.don.0000133155.04468.41

[b4] Tay F. R., Pashley D. H., Rueggeberg F. A., Loushine R. J. & Weller R. N. Calcium phosphate phase transformation produced by the interaction of the portland cement component of white mineral trioxide aggregate with a phosphate-containing fluid. J Endod. 33, 1347–1351 (2007). 17963961 10.1016/j.joen.2007.07.008

[b5] Charland T., Hartwell G. R., Hirschberg C. & Patel R. An evaluation of setting time of mineral trioxide aggregate and EndoSequence root repair material in the presence of human blood and minimal essential media. J Endod. 39, 1071–1072 (2013). 23880280 10.1016/j.joen.2013.04.041

[b6] Nekoofar M. H. *et al.* An evaluation of the effect of blood and human serum on the surface microhardness and surface microstructure of mineral trioxide aggregate. Int Endod J. 43, 849–858 (2010). 20636353 10.1111/j.1365-2591.2010.01750.x

[b7] Nekoofar M. H., Stone D. F. & Dummer P. M. The effect of blood contamination on the compressive strength and surface microstructure of mineral trioxide aggregate. Int Endod J. 43, 782–791 (2010). 20609024 10.1111/j.1365-2591.2010.01745.x

[b8] Oloomi K. *et al.* Evaluation of the effect of blood contamination on the compressive strength of MTA modified with hydration accelerators. Restor Dent Endod. 38, 128–133 (2013). 24010078 10.5395/rde.2013.38.3.128 PMC3761120

[b9] Kim Y., Kim S., Shin Y. S., Jung I. Y. & Lee S. J. Failure of setting of mineral trioxide aggregate in the presence of fetal bovine serum and its prevention. J Endod. 38, 536–540 (2012). 22414845 10.1016/j.joen.2011.12.005

[b10] Nekoofar M. H., Davies T. E., Stone D., Basturk F. B. & Dummer P. M. Microstructure and chemical analysis of blood-contaminated mineral trioxide aggregate. Int Endod J. 44, 1011–1018 (2011). 21718336 10.1111/j.1365-2591.2011.01909.x

[b11] Salem Milani A., Rahimi S., Froughreyhani M. & Vahid Pakdel M. Effect of Blood Contamination on Marginal Adaptation and Surface Microstructure of Mineral Trioxide Aggregate: A SEM Study. J Dent Res Dent Clin Dent Prospects. 7, 157–163 (2013). 24082987 10.5681/joddd.2013.025 PMC3779375

[b12] Moinzadeh A. T., Aznar Portoles C., Schembri Wismayer P. & Camilleri J. Bioactivity Potential of EndoSequence BC RRM Putty. J Endod. 2, 615–621 (2016). 26786381 10.1016/j.joen.2015.12.004

[b13] Cintra L. T. *et al.* Evaluation of subcutaneous and alveolar implantation surgical sites in the study of the biological properties of root-end filling endodontic materials. J Appl Oral Sci. 18, 75–82 (2010). 20379685 10.1590/S1678-77572010000100013 PMC5349029

[b14] Moretton T. R., Brown C. E. Jr, Legan J. J. & Kafrawy A. H. Tissue reactions after subcutaneous and intraosseous implantation of mineral trioxide aggregate and ethoxybenzoic acid cement. J Biomed Mater Res. 52, 528–533 (2000). 11007621 10.1002/1097-4636(20001205)52:3<528::aid-jbm11>3.0.co;2-9

[b15] International standards organization. Biological evaluation of medical devices. Tests for local effects after implantation. ISO 10993–10996 (2009).

[b16] Lenherr P. *et al.* Tooth discoloration induced by endodontic materials: a laboratory study. Int Endod J. 45, 942–949 (2012). 22506849 10.1111/j.1365-2591.2012.02053.x

[b17] Gomes-Filho J. E. *et al.* Evaluation of the tissue reaction to fast endodontic cement (CER) and Angelus MTA. J Endod. 35, 1377–1380 (2009). 19801233 10.1016/j.joen.2009.06.010

[b18] Kurita L. M. *et al.* Response of mice connective tissue to three different endodontic materials. Microsc Res Tech. 76, 311–315 (2013). 23335503 10.1002/jemt.22168

[b19] Sumer M., Muglali M., Bodrumlu E. & Guvenc T. Reactions of connective tissue to amalgam, intermediate restorative material, mineral trioxide aggregate, and mineral trioxide aggregate mixed with chlorhexidine. J Endod. 32, 1094–1096 (2006). 17055915 10.1016/j.joen.2006.05.012

[b20] Hammad H. M., Hamadah M. A. & Al-Omari W. M. Histological evaluation of rat tissue response to GMTA, Retroplast, and Geristore retrograde filling materials. Aust Endod J. 37, 18–25 (2011). 21418410 10.1111/j.1747-4477.2009.00195.x

[b21] Viola N. V. *et al.* Biocompatibility of an experimental MTA sealer implanted in the rat subcutaneous: quantitative and immunohistochemical evaluation. J Biomed Mater Res B Appl Biomater. 100, 1773–1781 (2012). 22821748 10.1002/jbm.b.32744

[b22] Yaltirik M., Ozbas H., Bilgic B. & Issever H. Reactions of connective tissue to mineral trioxide aggregate and amalgam. J Endod. 30, 95–99 (2004). 14977305 10.1097/00004770-200402000-00008

